# Cross‐protection interactions in insect pests: Implications for pest management in a changing climate

**DOI:** 10.1002/ps.7191

**Published:** 2022-10-18

**Authors:** Erika M. Bueno, Casey L. McIlhenny, Yolanda H. Chen

**Affiliations:** ^1^ Department of Plant and Soil Science University of Vermont Burlington VT USA; ^2^ Gund Institute for Environment University of Vermont Burlington VT USA

**Keywords:** cross‐protection, climate change, pest management, epigenetics, cross‐tolerance, cross‐talk

## Abstract

Agricultural insect pests display an exceptional ability to adapt quickly to natural and anthropogenic stressors. Emerging evidence suggests that frequent and varied sources of stress play an important role in driving protective physiological responses; therefore, intensively managed agroecosystems combined with climatic shifts might be an ideal crucible for stress adaptation. Cross‐protection, where responses to one stressor offers protection against another type of stressor, has been well documented in many insect species, yet the molecular and epigenetic underpinnings that drive overlapping protective responses in insect pests remain unclear. In this perspective, we discuss cross‐protection mechanisms and provide an argument for its potential role in increasing tolerance to a wide range of natural and anthropogenic stressors in agricultural insect pests. By drawing from existing literature on single and multiple stressor studies, we outline the processes that facilitate cross‐protective interactions, including epigenetic modifications, which are understudied in insect stress responses. Finally, we discuss the implications of cross‐protection for insect pest management, focusing on the consequences of cross‐protection between insecticides and elevated temperatures associated with climate change. Given the multiple ways that insect pests are intensively managed in agroecosystems, we suggest that examining the role of multiple stressors can be important in understanding the wide adaptability of agricultural insect pests. © 2022 The Authors. *Pest Management Science* published by John Wiley & Sons Ltd on behalf of Society of Chemical Industry.

## Graphical abstract

Insect pests encounter diverse sources of stress, which may contribute to their ability to rapidly adapt to natural and anthropogenic stressors. Cross‐protection may serve as an avenue for tolerance to multiple stressors. Here, we discuss mechanisms and uncover gaps in our understanding of cross‐protection interactions and its implications under climate change.

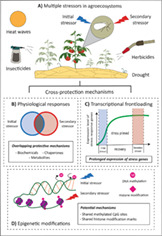

## 1 INTRODUCTION

**Figure 1:**
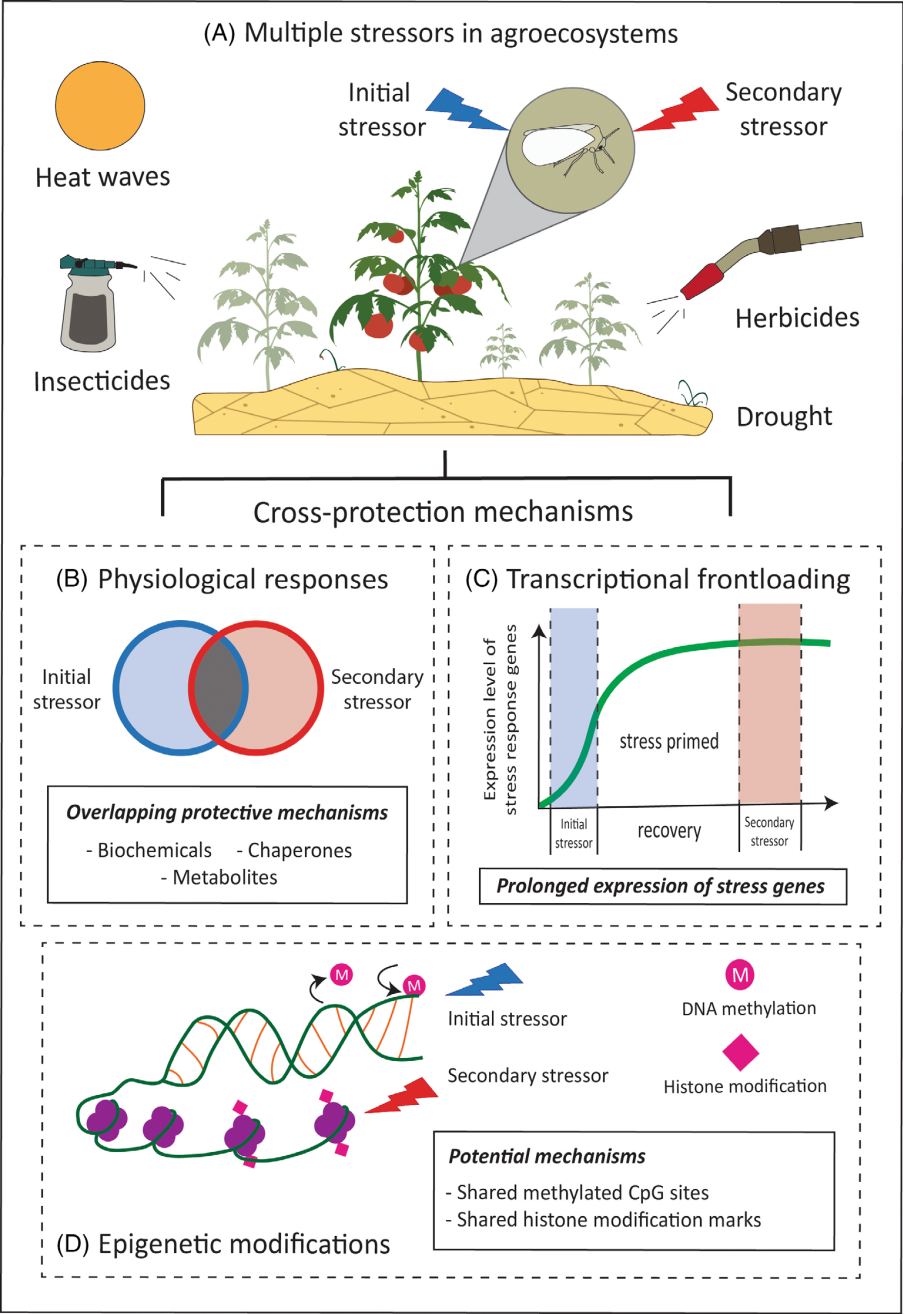
Examples of multiple stressors in agroecosystems and potential mechanisms for cross‐protection in insect pests. (A) Insect pests face multiple and diverse sources of stress in agroecosystems ranging from exposure heat waves, drought conditions, indirect contact with herbicides, and insecticide applications. (B) Cross‐protection between stressors may arise from an overlap in biochemical enzymes and the expression of stress inducible genes. (C) Initial stress may prolong the transcription of stress genes; long enough to confer protection against a secondary stressor. (D) Epigenetic modifications, such as DNA methylation and histone modifications, may activate the same methylated CpG sites or histone marks (i.e. acetyl groups), turning on/off the expression of genes associated with cross‐protection.

Agricultural insect pests directly threaten global food security by reducing annual crop production by 18–20% .^1^ To mitigate the impact of insect pests on agricultural production, a wide range of cultural, chemical and biologically based control measures are used to manage insect pests.^2^, ^3^ Despite these control measures, insect pests are able to persist in agroecosystems, largely due to their ability to rapidly respond to natural and anthropogenic stressors.^4^ Here, we define a stressor as a change in the environment (abiotic or biotic) that exerts a reduction in an organism's fitness and survival.^5^, ^6^ For insects, common forms of stress in agroecosystems arise from exposure to high temperatures, insecticides, herbicides, drought and environmental pollutants (Fig. 1(A)).^7^, ^8^, ^9^, ^10^, ^11^ While the ability of insect pests to persist in intensively managed agroecosystems depends on their capacity to tolerate multiple stressors, relatively little is known about the extent to which exposure to one form of stress can influence a pest's ability to tolerate subsequent stress. Given that environmental stress has long been considered a selective force in evolutionary biology, understanding how certain stressors interact to enhance or inhibit insect pest survival and performance will help improve our ability to make informed pest management decisions.

Environmental stress directly shapes phenotypic responses in insect pests, altering physiological, morphological and behavioral traits that allow insects to cope with highly variable conditions.^12^, ^13^, ^14^ As such, intensively managed agroecosystems combined with extreme environmental fluctuations may be an ideal crucible to drive the pace of adaptive stress responses in insect pests.^15^ Moreover, given that insect populations are projected to grow in response to rising temperatures, heavier use of agricultural chemicals will likely increase the possibility for stressor interactions.^16^, ^17^, ^18^ Ultimately, expanding our knowledge on the interactive effects of stress will improve our ability to predict changes in population size and devise strategies to sustainably manage insect pests under changing climatic conditions.^18^, ^19^, ^20^, ^21^, ^22^

Two processes that allow insects to thrive in highly dynamic environments are cross‐tolerance and cross‐talk, when exposure to one stressor enhances tolerance toward another stressor.^23^, ^24^, ^25^ While the phenotypic outcome of cross‐tolerance and cross‐talk are essentially the same (enhanced stress tolerance following initial stress), the mechanisms underlying each process differ. Under cross‐tolerance, individual stressors activate independent signaling pathways, resulting in shared physiological responses that provide overlapping protection.^23^ Whereas in cross‐talk, individual stressors activate shared signaling pathways that elicit distinct physiological responses that provide protection against each stressor.^23^ Despite these differences, cross‐tolerance and cross‐talk are difficult to distinguish from each other using empirical data. Hence, to account for both processes from here on, we use the term cross‐protection (as used by Rodgers *et al*.)^50^ to describe stressor interactions and mechanisms associated with enhanced stress tolerance due to prior stress exposure.

Although information surrounding the mechanistic basis of cross‐protection in insects is currently limited, previous studies have characterized shared protective responses that are induced following exposure to initial and secondary stress (Fig. 1(B)).^26^, ^27^, ^28^, ^29^, ^30^, ^31^, ^32^ These changes encompass a wide range of defensive strategies such as increases in antioxidants, metabolites, up‐regulation of molecular chaperones and detoxification enzymes, all of which help maintain the integrity and function of cellular processes important for survival.^26^, ^27^, ^28^, ^29^, ^30^, ^31^, ^32^ For example, in the malt fly, *Chymomyza costata*, enhanced freeze tolerance following exposure to drought stress was linked to the accumulation of proline, glutamine, and trehalose; three sugar metabolites with cryoprotective properties known to stabilize the function of macromolecules.^29^ In addition to biochemical responses, shared responses may also arise from changes in transcriptional regulation or through epigenetic modifications, molecular changes that regulate the expression of genes without altering the DNA sequence, potentially serving as another driving force behind cross‐protection (Fig. 1(D)).^33^, ^34^, ^35^, ^36^, ^37^, ^38^, ^39^

In this perspective, we review the evidence on cross‐protection mechanisms and provide an argument for its role in increasing tolerance to a range of natural and anthropogenic stressors. We conducted a literature search on stressor interactions to uncover trends in the use of stressor pairs and commonly used measures of physiological effects. Next, we illustrate what is currently known and expand on the idea of how transcriptional and epigenetic changes may act to promote cross‐protection in insect pests. While we mainly focus on stressor interactions related to cross‐protection, we note that not all stressor interactions result in enhanced tolerance. Stressor interactions may potentially induce cross‐susceptibility, where exposure to one stressor increases susceptibility toward a different stressor.^23^, ^24^ Additionally, we discuss the importance of multi‐stressor frameworks that integrate phenotypic and genomic approaches to identify cross‐protection mechanisms in insect pests. We then describe cases of cross‐protection between insecticides and elevated temperatures and discuss its implications on pest management in a changing climate. Lastly, we explain the value of using a multi‐stressor framework for quantifying stressor interactions and its corresponding effects on insect performance.

## 2 CROSS‐PROTECTION: AN AVENUE FOR TOLERANCE TO MULTIPLE STRESSORS IN AGROECOSYSTEMS

Protective responses like cross‐protection are advantageous as it allows organisms to survive in environments where multiple stressors co‐occur in space and time.^23^, ^24^, ^25^ Despite the propensity for insect pests to rapidly respond to stressful conditions, research on cross‐protection in insect pests remains limited. To evaluate the frequency of cross‐protection studies in insect pests, we searched Web of Science using a combination of keywords that are commonly associated with interactions among stressors, such as ‘cross‐tolerance’, ‘cross‐talk’, ‘cross‐resistance’, ‘cross‐protection’, ‘hormesis’, ‘pre‐treatment’, ‘preconditioning’, ‘pre‐exposure’, ‘priming’, ‘stress’, ‘stressor’, ‘interaction’, ‘insect’ and ‘insect pest’. We focused on experiments that used distinct initial and secondary stressors as treatments, while excluding studies that examined stressors of similar nature. Given the vast amount of literature on insect stress responses, we acknowledge that any resulting trends from our search is limited by our choice of keywords used to identify instances of cross‐protection interactions in insect pests. Nonetheless, our search generated 76 studies encompassing 130 experiments assessing cross‐protection between two or more stressors across a total of 56 insect species, including 52 agricultural pests (crop and stored grain) and four vector‐borne mosquito species.

Based on our search, 58% (76 out of 130) reported experiments found evidence of cross‐protection (Supporting Information Table S1). In addition, our search revealed trends in stressor choice, particularly among naturally occurring and anthropogenic forms of stress. Here, natural stressors are defined as stressors that occur in the environment in the absence of direct human influence (i.e. elevated temperatures), as opposed to anthropogenic stressors that are strictly man‐made. Among natural stressors, 48% of the experiments used elevated temperatures, desiccation and starvation as the initial treatment. Though limited to our choice of keywords, the focus on natural stressors likely reflects a growing interest in understanding how elevated temperatures coupled with dry conditions and periods of food scarcity promote protective responses against other forms of stress in agroecosystems, an issue that is expected to intensify with climate change.^16^

Experiments exclusively using pairs of anthropogenic stressors (25 out of 130; 19%) were less represented than experiments solely using natural stressors (53 out of 130; 41%). When examined in any order of combination, the number of experiments using natural and anthropogenic stressors were roughly similar, with 22 natural *versus* anthropogenic and 29 anthropogenic *versus* natural stressor combinations. Among anthropogenic stressors, insecticides, herbicides, and heavy metal pollutants constituted 31% (40 out of 130) of all experimental initial treatments. Such trends in usage may reflect growing efforts to investigate non‐target impacts of agrochemicals (herbicides and fungicides) on insect pest performance and overlapping responses involved in cross‐protection. For instance, pre‐exposure to herbicides has been shown to improve survival towards insecticides in the cotton bollworm (*Helicoverpa armigera*) and the tobacco cutworm (*Spodoptera litura*) via the up‐regulation of detoxification genes.^8^, ^40^ Similarly, exposure to fungicides have been shown to prime Colorado potato beetles (*Leptinotarsa decemlineata*) against insecticides through shared activation of detoxification enzymes.^41^, ^42^

**Figure 2:**
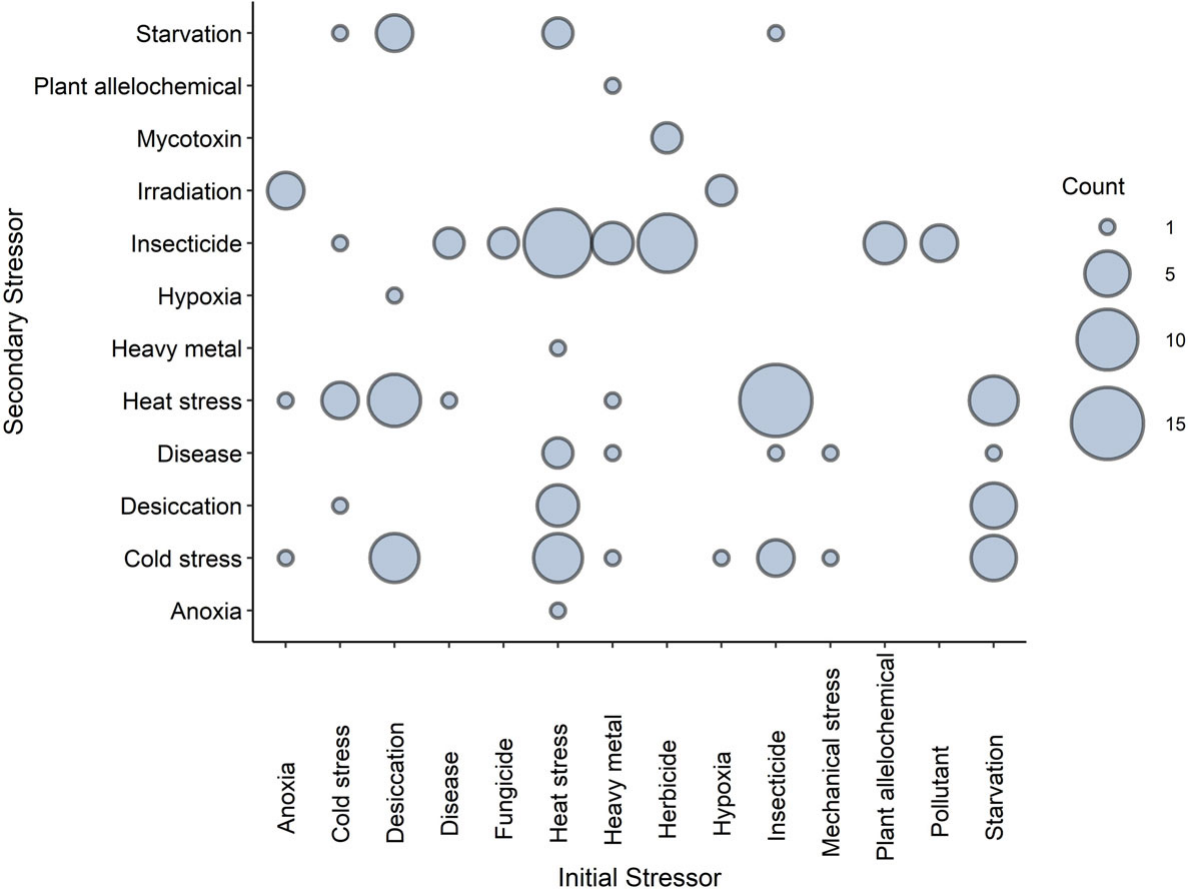
Number of cross‐protection experiments among different forms of stress in insect pests. The size of the count scale represents the number of experiments gathered from individual publications, including those that performed more than one experiment.

Most experiments (10 out of 12) in which elevated temperatures were used as an initial stressor, resulted in increased tolerance towards insecticides (Fig. 2). These results have been observed in a variety of insect pests, such as the serpentine leaf miner (*Liriomyza trifoli*), diamond back moth (*Plutella xylostella*), wheat aphid (*Sitobion avenae*), and brown planthopper (*Nilaparvata lugens*).^43^, ^44^, ^45^, ^46^ In the serpentine leaf miner, elevated temperatures improved survival against subsequent exposure to the insecticide (abamectin). However, when the order of the treatments was reversed (abamectin followed by heat), a reduction in survival was found, indicating that cross‐protection between heat and insecticides may depend on the order in which stressors occur in nature.^43^ Indeed, heightened susceptibility to insecticides when preceded by heat stress has been reported in other insects, presenting a common pattern in responses according to stressor order.^18^, ^47^, ^48^, ^49^ Though not entirely understood, differential responses based on stressor order may reflect a lack of shared mechanisms (cross‐tolerance) or the activation of common signaling pathways (cross‐talk), resulting in no cross‐protective responses. Due the complexity of stressor interactions, the sequence of exposure should always be taken into consideration when generalizing the effects of stressor pairs on insect performance.

We found through the literature search that a wide use of physiological metrics are used to identify cross‐protection, including acute measures of tolerance towards secondary stress and long‐term effects on insect performance. Acute measures of stress tolerance, such as mortality rates, recovery time and thermal limits (i.e. CT_min_/CT_max_) were commonly used to quantify immediate responses to initial and secondary stressors (Table S1). Though not as frequent, long‐term metrics such as developmental rates, fecundity, mating ability and total lifespan were also used to quantify less immediate impacts on insect performance (Table S1). While both metrics serve as indicators of the physiological state of individuals following stress, long‐term metrics are important for assessing recovery and impacts on fitness, and thus should be incorporated into experimental designs whenever possible.

Although numerous studies have identified cross‐protection in insect pests (Table S1), it is unclear whether cross‐protection phenotypes are heritable.^50^, ^51^, ^52^, ^53^ Nonetheless, emerging work on transgenerational stress effects in insects have uncovered beneficial outcomes for offspring generations, including increases in fecundity, population growth rates and tolerance to subsequent stressors.^45^, ^54^, ^55^, ^56^, ^57^, ^58^ As a result, transgenerational stress effects may provide a way for insects to respond rapidly to fluctuating environmental conditions. However, more work is needed to establish the heritability of cross‐protection before it can be considered a significant driving force of adaptive responses in insect pests.

## 3 WHAT DO WE KNOW ABOUT MECHANISMS UNDERLYING CROSS‐PROTECTION IN INSECTS?

The mechanisms that facilitate cross‐protection in insects are not entirely known. Yet, a variety of approaches have been used to investigate cross‐protection at distinct levels of organization, including biochemical and molecular responses at the gene and transcriptional level.^59^ The use of such approaches has provided insight into the processes that enhance tolerance toward secondary stressors in insect pests. In this section, we discuss the current knowledge around mechanisms associated with cross‐protection, drawing examples from our literature search and studies in insects and other taxa. We also consider the advantage of using comparative transcriptomics to identify the function of genes associated with cross‐protection, providing examples of studies that have implemented similar approaches. Lastly, we explain the gap in knowledge surrounding epigenetic modifications as a potential basis for cross‐protection outcomes.

### 3.1 Mechanisms associated with stress preconditioning via hormesis

Cross‐protection may arise due to stress preconditioning, when exposure to an initial stress induces a response that is maintained long enough to protect an organism from subsequent stress. In insects, stress preconditioning studies have demonstrated how prior exposure to stress can prepare insects for often more intense bouts of stress.^60^, ^61^, ^62^ These studies represent a large body of literature on stress acclimation, rapid thermal hardening, and hormesis, which have been previously reviewed.^62^, ^63^, ^64^, ^65^ Specifically, hormesis, which is defined by a biphasic response curve that describes stimulatory effects on performance at low doses and inhibitory effects at high doses, has been frequently described in insect pests.^64^, ^65^ For example, in green peach aphids (*Myzus persicae*), pre‐exposure to sublethal doses of imidacloprid resulted in increased survival under starvation stress.^54^ Given that low doses of stress have been shown to increase tolerance to other forms of stress, presumably via shared protective responses, cross‐protection can be considered a special case of hormesis.^64^ For this section, we focus on examples of hormesis that describe the influence of sublethal stress on tolerance to subsequent stressors.

#### 3.1.1 Biochemical responses to multiple stressors

Biochemical responses such as the induction of antioxidant enzymes and cryoprotectant biomolecules, are important physiological indicators of general stress responses, and have been examined in relation to cross‐protection in insect pests.^59^ Stressful conditions that trigger oxidative stress cause the accumulation of reactive oxygen species (ROS), which damage cellular structures and impair signaling pathways that ultimately lead to apoptosis.^66^, ^67^, ^68^ As a counter defense, insect cells launch a suite of protective responses, including the elevation of antioxidant enzymes that scavenge ROS and reduce cellular damage.^66^, ^67^, ^68^ Rises in antioxidant enzymes resulting from initial stress exposure appear to mediate tolerance toward subsequent stressors. For example, in the Caribbean fruit fly (*Anastrepha suspensa*), pre‐exposure to anoxic conditions increased antioxidant levels (superoxide dismutase and glutathione peroxidase), which in turn reduced oxidative damage under subsequent radiation exposure, improving tolerance and male mating performance.^27^ Likewise, pre‐exposure to hypoxic conditions in the cowpea bruchid (*Callosobruchus maculatus*) enhanced irradiation tolerance by reducing ROS levels.^69^ Decreases in ROS corresponded with low levels of citrate synthase, which is indicative of mitochondrial activity, suggesting that hypoxia facilitates irradiation tolerance by hindering mitochondrial respiration resulting in low ROS levels.^69^ Other biochemical compounds involved in cross‐protection include the production of sugar alcohols, trehalose and proline, which serve as sources of metabolic energy in response to heat and starvation and protect biomolecules from freeze damage following exposure to low temperatures.^28^, ^29^, ^70^

Genes associated with xenobiotic detoxification play a significant role in mediating tolerance to chemical stressors. In insects, detoxification genes, such as cytochrome P450s monooxygenases (CYPs), glutathione‐*S*‐transferases (GSTs) and esterases (ESTs), breakdown xenobiotic compounds and become overexpressed following exposure to insecticides, herbicides and plant defense compounds.^71^, ^72^ Therefore, it is not surprising that exposure to chemicals that activate the same detoxification enzymes, tend to give way to cross‐protection. For example, in the cotton bollworm (*H. armigera*), exposure to quercetin, a flavonoid‐based repellent, resulted in the up‐regulation of cytochrome P450s detoxification genes, which was correlated with higher tolerance to the pyrethroid insecticide, lambda‐cyhalothrin.^73^ These findings, along with others listed in Table S1, highlight the consistent role of detoxification enzymes in promoting cross‐protection between chemical stressors.

#### 3.1.2 Heat shock protein responses to multiple stressors

In insects, heat shock proteins (HSPs) help prevent the accumulation of misfolded proteins in response to a range of stressors including high and low temperatures, insecticides and hypoxic conditions.^74^ This versatile role makes HSPs a good indicator of stress tolerance, and as a result, have been largely implicated in facilitating hermetic responses to subsequent stress. For instance, in the brown plant hopper (*N. lugens*), pre‐exposure to mild doses of triazophos up‐regulated the expression of *Hsp70*, resulting in increased tolerance to elevated temperature, as indicated by higher in survival rates and increases in lethal mean time to 50% mortality (LT_50_) at 40 °C.^46^ It may be possible that the up‐regulation of *Hsp* genes, and perhaps other protective biochemical activity, remain expressed long after initial exposure to sublethal stress, which may explain how stress priming occurs in insects. Nevertheless, additional studies are needed to evaluate how long protective responses last after initial stress and whether prolonged expression exerts long‐term costs on fitness.

### 3.2 Transcriptional responses to multiple stressors

Shared transcriptional responses, where the same genes are activated in response to different stressors, is believed to be another process underlying cross‐protection. As such, RNA sequencing (RNA‐seq) approaches have been used to identify shared genetic responses that are associated with cross‐protection and cross‐susceptible phenotypes in insects.^10^, ^11^, ^42^ For example, single and combined exposure to abamectin and heat stress induced the transcription of 138 shared genes in *Liriomyza trifolii*.^11^ Individuals exposed to both heat and abamectin increased expression of stress genes including *Hsp70*, catalase (*Cat*) and *Cyp P450s*, providing a potential mechanistic explanation for the observed cross‐protection in *Liriomyza trifolii*.^11^ In *Leptinotarsa decemlineata*, treatment with either chlorothalonil (fungicide) or imidacloprid up‐regulated the expression of similar genes involved with metabolic detoxification and cellular defenses such as carboxylesterases, UDP‐glucuronosyltransferases, cytochrome P450s and Hsp70, implicating the possibility for cross‐protection between both agrochemicals.^42^

While the induction of shared transcriptional responses is one way in which cross‐protection may arise, other transcriptional processes may also be at play. For example, prolonged expression of constitutively expressed genes, can prepare organisms for subsequent stress exposure, reducing the requirement of induced responses to stress, a type of priming known as transcriptional frontloading.^75^, ^76^ While the concept of transcriptional frontloading has only been described in marine invertebrate studies, it serves as a relevant example of transcriptome‐wide responses that go beyond identifying shared genes in response to multiple stressors, thus improving our understanding of cross‐protection mechanisms in insects (Fig. 1(C)). For example, frontloading in heat‐acclimated marine intertidal amphipod, *Echinogammarus marinus*, displayed enhanced performance under hypoxic conditions, as demonstrated by a lower metabolic response.^76^, ^77^ Furthermore, overall gene expression patterns in heat acclimated individuals changed minimally compared to individuals acclimated to cold conditions, suggesting that frontloading may drive cross‐protection against hypoxia through the altered expression of constitutively expressed genes.^76^, ^77^ More studies are needed to determine whether transcriptional priming processes like frontloading mediate cross‐protection in insect pests.

#### 3.2.1 Comparative transcriptomics and functional genomics can lend insight into cross‐protection mechanisms

Comparative approaches using transcriptomic tools such as RNA‐seq enable us to quantify changes in gene expression levels in response to stressful conditions. Such approaches have been previously used to reveal processes underlying responses to single stressors, such as pathogen‐induced immune priming in bumblebees, *Bombyx mori*, heat stress tolerance in the less mulberry snout moth, *Glyphodes pyloalis*, and responses to plant secondary compounds in the soybean aphid, *Aphis glycines*.^77^, ^78^, ^79^ Despite its wide use in single stressor studies, the use of comparative transcriptomics within the insect pest cross‐protection literature remains limited. Applying omics approaches to multiple stressor studies will not only help characterize shared protective responses but open opportunities to better understand the physiological, genetic and metabolic basis for cross‐protection and cross‐susceptibility between stressors. For example, Jiang *et al*. applied a multi‐omics approach to understand how exposure to cadmium (Cd) influences spongy moth (*Lymantria dispar*) susceptibility to the fungus, *Beauveria bassiana*.^10^ In this study, transcriptomic and proteomic tools were used to quantify responses to *Beauveria bassiana* infections under low and high doses of Cd, which generated differentially expressed genes and proteins that were enriched for immune response pathways and metabolic detoxification mechanisms.^10^ Gene and protein level responses to combined stress were also found to be highly correlated to each other, providing a comprehensive and detailed view of the molecular pathways involved in cross‐susceptibility.^10^

As genomic sequencing becomes cheaper, access to annotated genomes is becoming increasingly available to insect researchers. In turn, functional tools such as RNA interference (RNAi) and CRISPR‐Cas9, which allow one to silence or knockout the expression of a gene of interest technologies, are becoming common techniques in non‐model insect studies.^80^, ^81^ For example, RNAi gene silencing has been successfully implemented to test the function of detoxification genes involved in insecticide resistance in wide range of insect pests.^82^, ^83^, ^84^, ^85^ Therefore, these tools provide an advantageous opportunity to uncover the function of genes involved in cross‐protection. For instance, the genes, *Hsp70* and *Argk* (arginine kinase), which activate protective responses against cellular stress, have been shown to mediate cross‐protection between insecticides and high temperature in the brown planthopper, *N. lugens*.^46^ By silencing the expression of *Hsp70* and *Argk* in planthoppers pre‐exposed to triazophos, Ge *et al*. demonstrated a significant reduction in LT_50_ at 40 °C when compared to untreated individuals [not treated with double‐stranded RNA (dsRNA)], suggesting that these genes play a key role in enhancing tolerance to both stressors.^46^ Ultimately, incorporating functional genomic tools in cross‐protection studies can provide a clearer understanding of genes involved in driving defensive responses to multiple stressors in insect pests.

### 3.3 Epigenetic modifications as potential drivers for cross‐protection

Epigenetic modifications such as histone modifications, DNA methylation and microRNAs (miRNAs) play a key role in reprogramming gene expression in response to environmental stimuli, altering insect phenotypes without changing the underlying DNA sequence.^39^, ^86^, ^87^ Histone modifications involve the addition of methyl or acetyl groups to the amino acid tails of histone proteins that condense DNA into chromatin, adjusting the overall accessibility of the DNA for transcription.^33^, ^39^ In eukaryotes, DNA methylation entails the addition or removal of methyl groups at CpG dinucleotides, resulting in the repression and activation of gene expression, respectively.^33^, ^39^ At the post‐transcriptional level, miRNAs regulate the expression of genes by targeting the 3′ untranslated regions (UTRs) of messenger RNAs (mRNAs) via complementary base pairing, thereby inhibiting the translation of mRNA into protein.^39^, ^88^ Together, epigenetic modifications appear to underlie the activation of inducible genes in response to external stress.^89^ For example, in *Diploptera punctata*, exposure to elevated temperatures resulted in higher levels of global DNA methylation and higher proportions of methylated cytosines in *Hsp70*, indicating that methylation can modulate the expression of stress responsive genes.^88^

While there is growing evidence that epigenetic modifications respond to stressful conditions, the study of the relationship between stress and epigenetic modifications is a newly emerging field in insect pest research.^89^, ^90^, ^91^, ^92^ In this section, we highlight the findings of previous epigenetic studies in insect pests that have measured responses to single stressors. We specifically focus on the two most studied epigenetic modifications in insects, histone modifications and DNA methylation, and describe each process in detail and within the context of stress responses.

#### 3.3.1 Histone modifications

Histone modifications, which involve the addition of acetyl or methyl group to histone proteins, regulate the expression of genes by altering DNA accessibility to transcription factors that initiate transcription by RNA polymerase II.^93^, ^94^ Although studies examining how stressful conditions influence histone modifications are limited for insect pests, the expression of enzymes associated with histone modifications have been investigated in response to thermal and oxidative stress in the red flour beetle, *Tribolium castaneum*.^91^ In this experiment the expression levels of class I histone deacetylases (TcHDACs), which catalyze the removal of acetyl groups from histones and repress gene transcription, were generally up‐regulated in response to cold and paraquat (herbicide) exposure and down‐regulated in response to heat stress.^91^ While these findings indirectly demonstrate that histone acetylation is responsive to abiotic stress, more research at the histone level is needed to confirm whether histone modifications act as drivers for cross‐protection in insect pests. Accomplishing this would require the identification of specific histone modifications (i.e. acetylation or methylation) that regulate common transcriptional responses between stressors.

Chromatin accessibility is also regulated by Polycomb (PcG) and Trithorax (TrxG), proteins that are responsive to abiotic stress in insects.^95^ PcG and TrxG proteins regulate histone modifications by adding and removing methylation, ubiquitylation and acetylation marks, thereby modulating gene expression.^96^ TrxG proteins allow the chromatin to be relaxed and available for transcription, which is associated with higher stress tolerance.^96^ For instance, TrxG proteins have been shown to directly enhance insect tolerance to food shortage and oxidative stress, but at a cost of insect longevity.^95^ In *Drosophila melanogaster*, PcG and TrxG proteins have been associated with the activation of stress inducible genes in response to desiccation, as indicated by an enrichment of H3K27me3 and H3K4m3 marks upstream of stress genes, suggesting that histone modifications play a role in regulating the expression of genes in response to environmental stress.^97^ More investigation is needed to test the function of PcG and TrxG in other insects, but the activity of these proteins may serve as a potential indicator for changes in histone modifications following exposure to multiple stressors.

#### 3.3.2 DNA methylation

DNA methylation has been shown to modulate development, phenotypic plasticity and responses to environmental stress in several insect groups.^92^, ^98^, ^99^, ^100^, ^101^ In insects, DNA methylation in promoter regions is associated with gene silencing, whereas methylation in protein‐coding regions (exons) results in active gene expression.^102^ The maintenance and establishment of methyl groups (*de novo*) is mediated by DNA methyltransferase (DNMT) enzymes that catalyze the addition of methyl groups to CpG sites.^100^, ^102^ In *de novo* methylation, insect DNA methyltransferases appear to respond to temperature stress. In the northern armyworm (*Mythimna separata*), inhibition of *Dnmt1* via RNAi negatively impacted larval development under elevated temperatures.^103^ Comparably, RNAi silencing of *Dnmt3* reduced heat and cold tolerance in the white fly (*Bemisia tabaci*), indicating that *de novo* methylation is associated with thermal stress responses.^90^

Although DNA methylation may drive rapid responses to environmental stress, only a handful of insect studies have explored how DNA methylation is impacted by exposure to single stressors, let alone multiple stressors. Many of these studies have explored how agricultural chemicals influence global levels of DNA methylation. For instance, the loss of methylation in genes coding for esterase enzymes was associated with an increase in susceptibility to insecticides in the peach potato aphid (*Myzus persicae)*, suggesting that methylation may facilitate the up‐regulation of detoxification genes.^104^ More recently, in *Leptinotarsa decemlineata*, parental exposure to imidacloprid decreased global methylation levels in un‐exposed offspring, which was linked to the de‐methylation of CpG sites within genes coding for cytochrome P450s, indicating that methylation may act over generations to improve insecticide tolerance.^92^ In another study, parental exposure to fungicides reduced mosquito sensitivity to insecticides in offspring generations, a trait that was correlated with increases in global DNA methylation in the Asian tiger mosquito (*Aedes albopictus*).^98^ Patterns of differential methylation have also been studied in response to thermal stress, revealing differences in methylation levels among genomic regions and genes following high temperature exposure.^105^ For instance, in a comparative analysis among silkworm (*Bombyx mori*) strains, resistance to high temperatures were linked to high levels of CpG methylation near transcriptional start sites and exons, and within genes enriched for RNA binding and transport.^105^ While we can only infer epigenetic mechanisms from general single stressor studies, different forms of stress may induce overlapping methylation patterns that regulate the activation of signaling pathways involved in cross‐protection. Consequently, experiments using multiple stressors are required to determine if single and combined stressors influence the presence/absence of methylated CpG sites and whether such changes correlate with the expression of shared genes between stressors.

## 4 CROSS‐PROTECTION BETWEEN INSECTICIDES AND ELEVATED TEMPERATURES AND ITS IMPLICATION WITH CLIMATE CHANGE

Increased temperatures associated with climate change are projected to increase population growth and metabolic rates in insect pests, resulting in devastating crop losses and increased insecticide use.^15^, ^16^, ^17^ As intensive insecticide use is a major driver of resistance, temperature increases may impact evolution rates in insect pests, leading to surges in resistance cases around the world. Although faster rates of evolution have not been documented in the field, they have been documented in the laboratory. For instance, Schneider *et al*. conducted a selection experiment to investigate how elevated temperature and atmospheric CO_2_ impact life history and performance traits in the Mexican bean weevil (*Zabrotes subfasciatus*).^106^ After ten generations, weevils under simulated conditions developed faster than the controls, suggesting that climatic conditions associated with climate change can enhance insect performance traits.^106^

Furthermore, higher mean temperatures or frequent heat waves may negatively impact the efficacy of insecticides, making them less toxic to insects.^21^, ^107^, ^108^ Temperature induced tolerance to insecticides has been observed in a handful insect pests and mosquito vectors. In the diamondback moth (*P. xylostella*), warm temperature pre‐treatments enhanced tolerance toward avermectin and fipronil, as demonstrated through higher survival rates and median lethal dose (LD_50_) values, respectively.^44^, ^58^ Cases of improved tolerance towards insecticides following high‐temperature treatments have also been reported in the wheat aphid (*Sitobion avenae*), brown planthopper (*N. lugens*) and the wheat weevil (*Sitophilus granarius*).^42^ Taken together, these studies underlie a common theme; elevated temperatures facilitate enhanced responses toward insecticide stress, allowing insects to survive challenging conditions. However, when stressor order is reversed, pre‐exposure to insecticides can reduce high temperature tolerance in insect pests, indicating that, depending on the sequence in which stressors occur, insecticide toxicity may be exacerbated under warmer conditions.^7^, ^18^, ^43^, ^47^ Hence, as understanding how climate‐related stressors interact with insecticides to promote cross‐protection or cross‐susceptibility in insect pests will be vital for predicting how climate change influences outbreak risk and geographical expansions into novel areas.

## 5 THE IMPORTANCE OF A MULTI‐STRESS FRAMEWORK FOR MEASURING STRESS RESPONSES IN INSECT PESTS

Current understanding of how stress impacts changes in genetic, physiological and behavioral responses has been profoundly shaped by studies that use single stress frameworks, where the effects of one stressor on organismal performance are contrasted against a control group.^52^ As such, single stressor studies continue to be foundational to our understanding of generalized responses to a wide range of stressors.^109^, ^110^, ^111^, ^112^ Yet, despite its usefulness, single stress frameworks may not be as relevant to the study of insect pests that are regularly exposed to a combination of physical, biotic and chemical stressors that can interact in ways that enhance stress tolerance.^113^, ^114^, ^115^ Such interactions can result in additive and non‐additive effects on insect performance and survival.^24^, ^25^, ^52^ Additive effects occur when the combined effect of each stressor is equal to the sum of each individual stressor alone.^52^ Whereas non‐additive effects occur when the combined effect of each stressor is lower or greater than the additive effect, resulting in antagonistic and synergistic responses, respectively.^52^ To this end, non‐additive effects on insect pest performance can be used to identify instances of cross‐protection (a form of antagonism) or cross‐susceptibility (a form of synergism) between stressors.^24^, ^52^ Here, we discuss the value of multi‐stress frameworks for identifying the interactive effects between stressors using molecular and epigenetic studies, point out key factors that influence cross‐protection experiments, and consider the limitations associated with multiple stressors studies.

### 5.1 Full factorial designs reveal interactive effects between stressors

To identify cross‐protection between stressors, an ideal experimental framework would test the direct effects of each individual stressor along with the combined effect of both stressors. Full factorial designs, where each stressor and corresponding levels are crossed with one another, offer an effective way to achieve this design, by testing for interactions between multiple stressors and assessing their impact on insect performance and survival.^116^, ^117^ However, for this design to be effective, statistical contrasts between combined stress treatments and individual stress treatments are required to identify non‐additive effects, including antagonistic interactions associated with cross‐protection. Inconsistent comparisons of stressor effects within full‐factorial designs may lead to missed opportunities for identifying non‐additive interactions.^52^ Thus, we encourage the use of full factorial designs within a multi‐stress framework and the use of statistical models that incorporate all combinations of stressor comparisons to identify non‐additive effects.

Multi‐stress frameworks can also be applied toward investigating genetic and transcriptomic mechanisms underlying responses to stress. Comparative approaches that distinguish unique *versus* shared genes that are differentially expressed under exposure to single or combined stress can help uncover the degree to which transcriptomic are shared between stressors.^80^ Shared genes can be further analyzed to identify signaling networks and gene ontology (GO) terms associated with stress‐inducible molecular responses.^80^ Additionally, multi‐stress frameworks can be utilized to identify interactions between stressors (additive *versus* non‐additive) at the level of gene expression.^80^, ^81^ This approach was recently used to identify interactions between chlorpyrifos and high temperatures on patterns of gene expression in *Culex pipiens*, in which the authors found evidence for interactions (additive *versus* non‐additive) on expression levels, with antagonistic up‐regulation being the most prevalent among stress genes.^117^

### 5.2 Perspectives on epigenetic studies using multiple stressors

To fully understand the adaptive toolkit that allows insect pests to thrive under stressful conditions, it is essential to evaluate the role of epigenetic modifications in mediating tolerance to both single and secondary stressors (Fig. 1(D)). By using a multi‐stressor approach, high‐throughput sequencing approaches, such as whole‐genome sequencing of bisulfite‐treated DNA (WGBS) and chromatin accessibility sequencing (ATAC‐seq), are excellent methods and tools to measure genome‐wide patterns of DNA methylation and histone modifications.^118^, ^119^, ^120^ Preferably, multi‐stressor epigenomic approaches should be performed at the tissue level using as many biological replicates per treatment as possible to reduce variability from individuals and to increase the resolution of tissue‐specific differences in responses. Differential epigenetic responses should also be complemented with other ‘omics approaches like transcriptomics or proteomics to test for the differences in epigenetic signals with transcript/protein expression data gathered from multi‐stressor experimental designs. Lastly, functional studies using gene silencing tools should be carried out to confirm the function of stress‐responsive genes in conferring cross‐protection to multiple stressors.

To evaluate whether epigenetic modifications influence cross‐protection, multi‐stressor experiments can be carefully designed to evaluate differences in methylated CpG sites between initial stress exposure, subsequent stress exposure, and a control (‘no stressor’) treatments. To this end, bioinformatic analyses of differentially methylated CpG sites can be mapped to genes, allowing one to characterize epigenomic profiles in response to single and subsequent stress exposure. For instance, the identification of overlapping and uniquely methylated sites between single and subsequent stress treatments can allow one to determine whether the same CpG sites are responding to different stressors. With the use of transcriptomic datasets originating from the same experimental design, correlations between differential methylated sites and transcripts can be constructed to test the role of methylation in repressing or activating genes involved in cross‐protection. Emerging tools like CRISPR‐guided DNA methylation editing, could also be used to verify the functionality of methylation at individual CpG sites.^120^ In sum, the use of epigenomic sequencing tools combined with expression and functional studies will allow for a better understanding of how epigenetic modifications drive protective responses to stress, providing a way to identify molecular drivers that confer cross‐protection to stress in insect pests.

### 5.3 Essential factors to consider when studying cross‐protection

The manifestation of cross‐protection depends on several factors, including the intensity, duration, and frequency of each stressor. Considering that severe stress exposure can negatively impact recovery and survival, the intensity of the initial stressor (in magnitude or duration) is determinant of whether cross‐protection will occur.^52^, ^121^ For instance, prolonged activation of defensive mechanisms may not become activated if the intensity of the initial stressor is not strong enough to induce a protective response.^52^ Conducting preliminary assessments with additional stressor intensities may be one solution, however, including multiple variables can make a simple experimental design increasingly complicated and may require justifying the relevancy of stressor intensities to actual field conditions. Narrowing down the appropriate stressor intensities can be achieved by using field relevant levels/concentrations of a stressor, especially if realistic ranges are known for the stressors of interest. The use of field‐relevant stressor intensities provide greater realism for predicting if stressors interact to influence insect performance in agroecosystems.

In cross‐protection studies, field relevant intensities have been considered for a broad range of agricultural stressors, including concentrations of heavy metals found on contaminated crops, the application of field recommended as well as sublethal insecticide doses, temperature regimes that simulate heat waves, and periods of food scarcity.^18^, ^48^, ^54^, ^122^ When studying environmental stressors associated with climate change, using projected intensities may help narrow down the number of variables needed for an experiment, while providing a realistic prediction on the interactive effects of multiple stressors. For example, Delnat *et al*.^18^ used existing levels of chlorpyrifos and projected mean temperature for 2100 under the 4 °C warming scenario to explore the interactive effects between elevated temperature and insecticides on mosquito survival.^18^, ^117^, ^123^

Another factor worth considering is the duration of time between the initial and secondary stressor, also known as the recovery period.^50^, ^121^ At one extreme, if the initial and secondary stressors overlap or occur immediately after one another, the interactive effects of both stressors may negatively impact an individual's ability to recover, limiting the possibility for cross‐protection. On the other extreme, if the initial and secondary stressor occur too far apart from each other, then cross‐protection is less probable as defenses against the initial stressor may have already subsided, and the individual is fully recovered. In this case, individual protective responses to each stressor, as opposed to overlapping responses, is expected. Overlapping protective mechanisms are more likely when the secondary stressor occurs shortly after the initial stressor, in other words, while the defensive responses of the initial stressor are activated.^51^ For example, mealworms (*Alphitobius diaperinus*) exposed to dry conditions became more tolerant to low‐temperatures but only when individuals recovered for half a day (12 h) between exposures.^7^ Conversely, when individuals were not allowed to recover in between stressors, no cross‐protection between desiccation and low temperature was observed, suggesting that the activation of shared protective mechanisms may be dependent upon the duration of the initial recovery phase.^7^ Potentially, protective responses may be continuously expressed long after the initial stress has ceased, resulting in increased tolerance to subsequent stress. Activation of protective responses long after initial stress has been demonstrated in *Drosophila melanogaster*, where flies pre‐exposed to multiple bouts of mild heat stress at a young age displayed higher levels of Hsp70 in response to higher temperatures later in life.^124^, ^125^ Further characterization of insect stress responses over time is required to evaluate how often biochemical/molecular responses are continuously activated or diminished over time following exposure to stress.

Cross‐protection and stress tolerance in general, may be influenced by how frequent stressors are presented in both field conditions and experimental settings. For instance, repeated exposure to cold temperatures, which simulate natural winter conditions for eastern spruce budworms (*Choristoneura fumiferana*), had a strong negative effect on survival than individuals subjected to prolonged and varying levels of low‐temperature exposure, highlighting the influence of varying exposure regimes on stress tolerance.^121^ Altogether, it is essential to consider not only the relevancy of stressors to actual agricultural conditions, but also the intensity, frequency and timing in which initial and secondary stressors are presented as these factors have been shown to strongly influence stress responses.

### 5.4 Limitations of multi‐stressor frameworks

Multi‐stress frameworks not only offer a practical way to identify interactions among stressors but can serve as an effective approach to characterize the molecular response to single and combined responses to stress. However, increasing the number of stressor combinations can challenge the feasibility of studies especially when varying levels of stress intensities, exposure durations and stressor frequency are incorporated into one experiment.^24^, ^52^ Furthermore, more stressor combinations may result in the saturation of stress responses if the combined effect of all stressors induce the same mechanism or may accelerate synergistic responses if the combined stressor effect is synergistic by nature.^52^ Therefore, we advise limiting the number of stressors to a manageable degree whenever possible and narrowing down stressor combinations by focusing stressors that are likely to interact and drawing on information from existing studies in closely related taxa (if available).

## 6 SUMMARY

Cross‐protection may play an imperative role in the persistence of insect pests in agriculture. Characterizing the mechanisms that drive insect pest's resilience in agroecosystems has important implications for investigating adaptive and phenotypically plastic responses to stressful conditions. Despite extensive research on single stress studies in insects, information regarding the impacts of multiple stressors and underlying processes that drive cross‐protection remain limited in insect pests. Given this gap in research, cross‐protection should be tested using a multi‐stressor framework that incorporates physiological, transcriptomic and epigenomic tools to identify key processes that facilitate protection against multiple stressors. To this end, it is also critical to link genomic and epigenomic responses to phenotypic traits as these traits provide more biologically relevant information. With the effects of climate change underway, it is increasingly pressing to improve our knowledge around the interactions between climate‐related stressors and insecticides, particularly within the context of insecticide resistance.

## CONFLICT OF INTEREST

The authors declare no conflict of interest.

## Supporting information


**Table S1.** List of cross‐protection experiments on agricultural insect pests and mosquito vectors. The physiological response/effect measured in each study are listed with the corresponding metric that was used to determine cross‐protection along with the reference. Critical thermal temperature; CT_max_/CT_min_, heat knockdown time (HKDT); chill coma recovery time (CCRT); lethal dose/time to mortality (LD_50_/LT_50_); heat shock protein (HSP70)

## Data Availability

Data sharing is not applicable to this article as no new data were created or analyzed in this study.
